# Malignant peripheral nerve sheath tumour of the oesophagus: a case report

**DOI:** 10.1186/s40792-020-00954-2

**Published:** 2020-07-31

**Authors:** Kento Tomizawa, Tatsuya Miyazaki, Arisa Yamaguchi, Ryoya Honda, Marie Hoshino, Mitsuhiro Yanai, Yohei Miyamae, Ryo Kurosaki, Hisashi Shimizu, Kazuhisa Arakawa, Munenori Ide

**Affiliations:** 1 Maebashi Red Cross Hospital Surgery Department, 389-1, Asakura-machi, Maebashi-shi, Gunma 371-0811 Japan; 2 Maebashi Red Cross Hospital Pathology Department, 389-1, Asakura-machi, Maebashi-shi, Gunma 371-0811 Japan

**Keywords:** FDG-PET, Ki67 index, surgical treatment

## Abstract

**Background:**

Malignant peripheral nerve sheath tumour (MPNST) is a very rare disease, and its pathogenesis is unknown. There are few reports of MPNST of the oesophagus. We report a case of an MPNST that was diagnosed and resected.

**Case presentation:**

A 30-year-old female presented with dysphagia. She had been aware of the dysphagia approximately 6 months before presentation. The chest X-ray showed shadows in the right mediastinum. Barium fluoroscopy revealed a semicircular raised lesion in the lower oesophagus. Upper gastrointestinal endoscopy revealed a type 1 oesophageal tumour centred on the posterior wall 26–35 cm from the incisors. The surface was ulcerated, and the tumour was exposed. The affected area showed no iodine uptake. The EUS showed an isoechoic mass. The CT scan showed a mass of 71 × 61 × 55 mm in the beginning of the lower oesophagus with low density mass and swelling of the right recurrent nerve lymph node to 12 mm. On FDG-PET, the tumour showed an SUVmax of 11.05, and no abnormal accumulation was found in lymph nodes or other organs. The MRI showed a hyperintense mass on the T2WI, which had prolonged contrast enhancement, and no findings of invasion into surrounding tissue were found. The patient underwent right thoracotomy and open thoracic oesophagectomy. The affected lymph node was tumour negative by rapid pathological diagnosis during the operation. Histologically, spindle cells with different-sized nuclei were mixed throughout the tissue. Some regions showed nuclear polymorphism or a storiform pattern, and locally, there were approximately 7 mitoses/10 HPFs. The margin was relatively clear, but spindle-shaped tumour cells infiltrated the surrounding interstitium and basal myoepithelium, and the patient was diagnosed with MPNST. In this case, the postoperative course was good, and 16 months after the operation, the patient is currently under observation at the outpatient stage without recurrence.

**Conclusions:**

MPNST in the oesophagus is a relatively rare disease. Diagnosis before treatment is sometimes difficult, but the prognosis is good if radical resection is possible.

## Background

Malignant peripheral nerve sheath tumour (MPNST) is the sixth most common type of soft-tissue sarcoma, accounting for approximately 5 to 10% of cases [1–3]. Although its exact cellular origins remain unclear, most MPNSTs arise in association with a peripheral nerve and are hypothesized to be of neural crest origin [3, 4]. Gastrointestinal malignant peripheral nerve sheath tumours are rare, and malignant peripheral nerve sheath tumours of the oesophagus are even rarer. Sarcomas of the oesophagus are rare, representing 0.1–1.5% of all oesophageal tumours. There are few reports of MPNST of the oesophagus. We report a case of MPNST that was diagnosed and resected.

## Case presentation

A 30-year-old female presented with dysphagia. She was obese, with a height of 157.8 cm and a weight of 86.8 kg. Her body mass index (BMI) was 34.9.

She had been aware of the dysphagia for approximately 6 months before presentation. During a medical check-up for work, a chest X-ray showed a shadow on the mediastinum, so the patient visited a doctor. The chest CT showed a 55-mm circular shadow. Three years ago, an upper GI endoscopy revealed a raised lesion in the left anterior wall of the oesophagus. At that time, the lesion was 1/3 of a circle and approximately 25 mm in size. This lesion was diagnosed as leiomyoma and was monitored. The patient had no medical history and no specific mention of a family medical history.

**Fig. 1:**
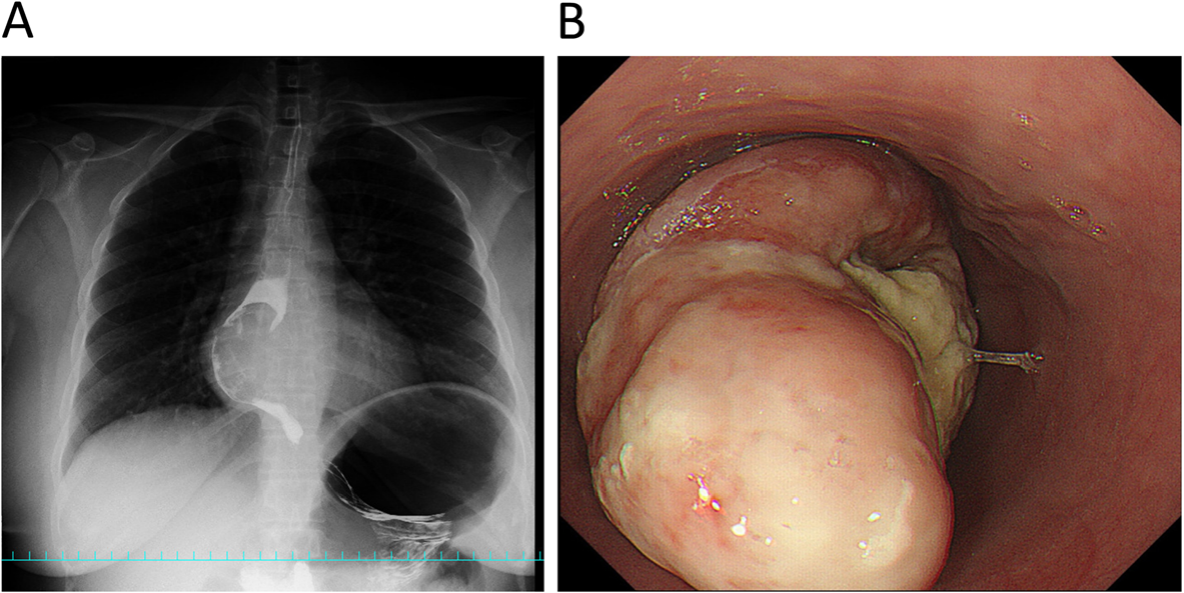
**a** Upper gastrointestinal endoscopy revealed a type 1 oesophageal tumour centred on the posterior wall 26–35 cm from the incisors. The surface was ulcerated, and the tumour was exposed. **b** Upper gastrointestinal imaging revealed a semicircular raised lesion in the lower oesophagus

**Fig. 2:**
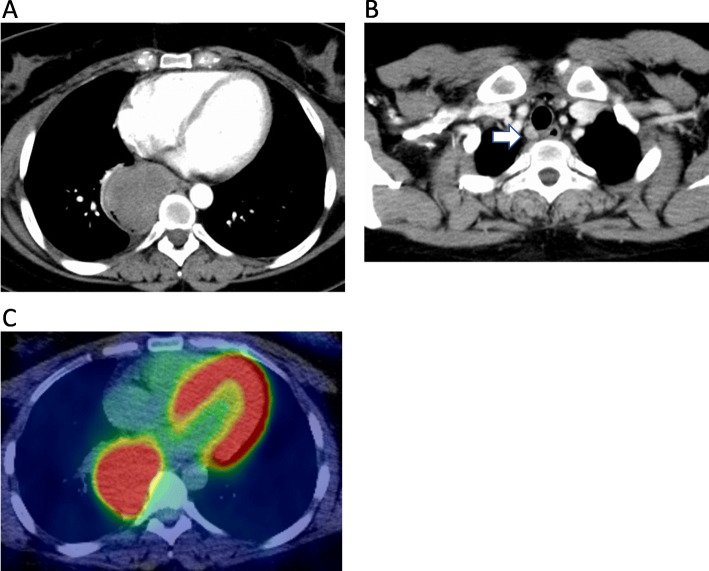
**a** The CT scan showed a mass of 71 × 61 × 55 mm in the beginning of the lower oesophagus with poor differentiation and progressive contrast enhancement. **b** The white arrow indicates swelling of up to 12 mm of the right recurrent nerve lymph node on the CT scan. **c** On FDG-PET, the tumour showed an SUVmax of 11.05, and no abnormal accumulation was found in lymph nodes or other organs. FDG-PET, 18F-fluorodeoxyglucose positron emission tomography

**Fig. 3:**
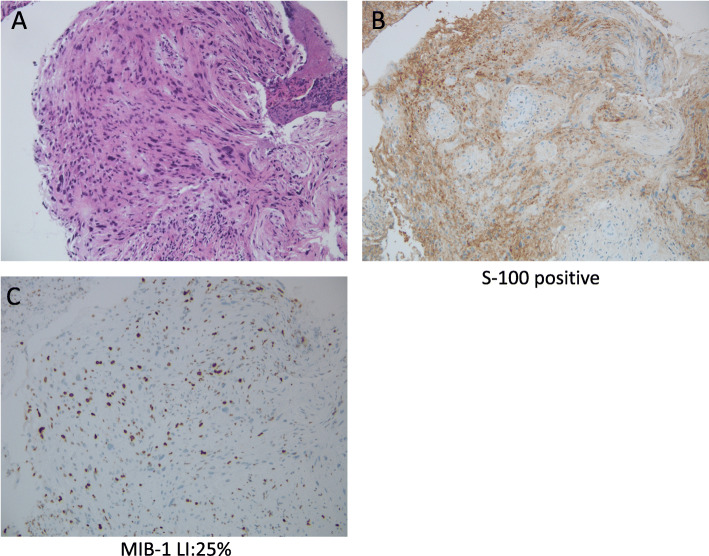
**a** Histopathological findings of the biopsy sample. The pathological specimen revealed that spindle cells with different-sized nuclei were mixed throughout the tissue. **b** Immunostaining showed S-100-positive staining. **c** Immunostaining showed MIB-1-positive staining. The MIB-1 LI was approximately 25%

Blood tests showed a slight increase in white blood cell count and CRP. CEA, CA 19-9, NSE, SCC, and p53 levels were within the reference ranges. The chest X-ray showed shadows in the right mediastinum (Fig. 1a). Barium fluoroscopy revealed a semicircular raised lesion in the lower oesophagus (Fig. 1a). Upper gastrointestinal endoscopy revealed a type 1 oesophageal tumour centred on the posterior wall 26–35 cm from the incisors (Fig. 1b). The surface was ulcerated, and the tumour was exposed (Fig. 1b). The tumour was hard and white. The affected area showed no iodine uptake. The EUS showed an isoechoic mass. The CT scan showed a mass of 71 × 61 × 55 mm in the beginning of the lower oesophagus with low density mass (Fig. 2a) and swelling of the right recurrent nerve lymph node to 12 mm (Fig. 2b). On FDG-PET, the tumour showed an SUVmax of 11.05, and no abnormal accumulation was found in lymph nodes or other organs (Fig. 2c). The MRI showed a hyperintense mass on the T2WI, which had prolonged contrast enhancement, and no findings of invasion into surrounding tissue were found. The endoscopic biopsy showed cascading spindle cells with different-sized nuclei (Fig. 3a), and immunostaining showed S-100 (+) (Fig. 3b), keratin (−), CD34 (−), and desmin (−) staining. The MIB-1 LI was approximately 25% (Fig. 3c), which led to the diagnosis of malignant schwannoma.

**Fig. 4:**
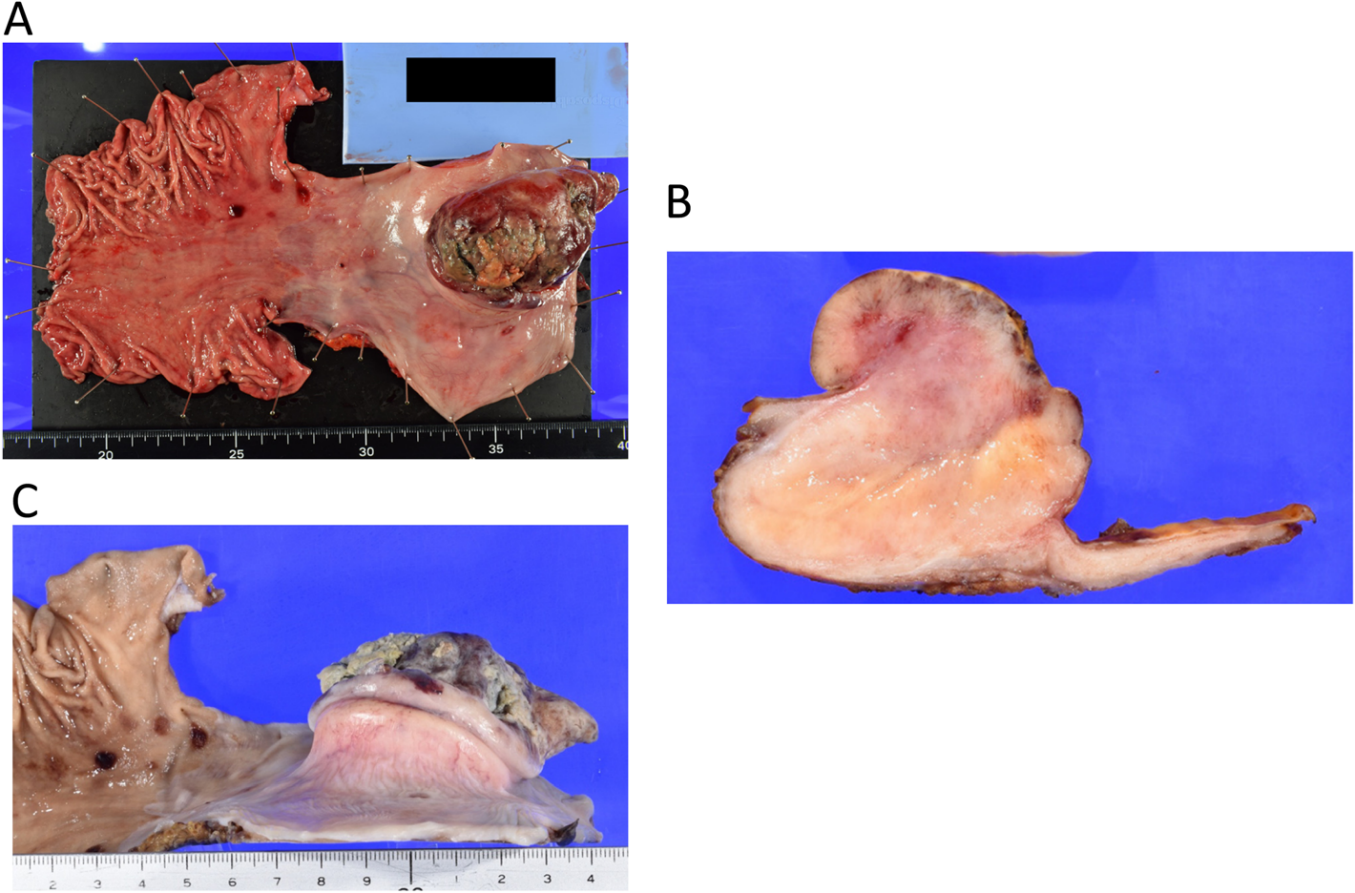
**a** Macroscopic findings. The resected tumour was 75 × 45 × 45 mm in size. **b** A large bulging lesion with a degenerative necrosis was observed in the neck. **c** The raised part of the tumour was covered with normal mucosa.

We performed right thoracotomy and open thoracic oesophagectomy. There were no pleural effusions or adhesions in the pleural cavity. The swollen right recurrent nerve lymph node was negative for tumours by rapid pathological diagnosis during the operation. We dissected all of the thoracic oesophagus with tumours to achieve radical resection. We placed a gastric tube through the intrathoracic route for reconstruction. Intrathoracic oesophagogastric anastomosis was performed using the circular stapling technique. The resected tumour was white, hard, and 75 × 45 × 45 mm in size (Fig. 4). A large bulging lesion in the neck with degenerative necrosis was observed in the specimen. The raised part of the tumour was covered with normal mucosa (Fig. 4c).

**Fig. 5:**
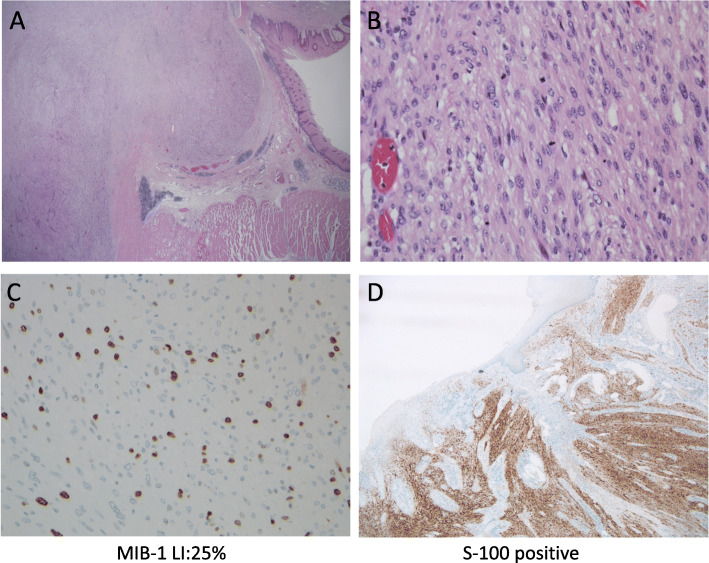
**a**, **b** Histopathological findings. The pathological specimen revealed that spindle cells with different-sized nuclei were mixed throughout the tissue. Some regions showed nuclear polymorphism or a storiform pattern, and locally, there were approximately 7 mitoses/10 HPFs. The margin was relatively clear, but spindle-shaped tumour cells infiltrated the surrounding interstitium and basal myoepithelium, and the patient was diagnosed with malignant peripheral nerve sheath tumour (MPNST). **c** Immunostaining showed MIB-1-positive staining. The MIB-1 LI was approximately 25%. **d** Immunostaining showed S-100-positive staining.

Histologically, spindle cells with different-sized nuclei were mixed throughout the tissue (Fig. 5a). Some regions showed nuclear polymorphism or a storiform pattern, and locally, there were approximately 7 mitoses/10 HPFs (Fig. 5b). The MIB-1 LI was approximately 25% (Fig. 5c). The margin was relatively clear, but spindle-shaped tumour cells infiltrated the surrounding interstitium and basal myoepithelium, and the patient was diagnosed with MPNST (Fig. 5a, b). Immunostaining showed S-100 (+) (Fig. 5d), CD34 (−), and HMB-45 (−) staining. These results led to a diagnosis of MPNST. In this case, the postoperative course was good, and 16 months after the operation, the patient is currently under observation at the outpatient stage without recurrence.

## Discussion

In PubMed, a medical publication database, there are 16 case reports of oesophageal MPNST, including this case [5–11] (Table 1). The onset age was 27–76 years, with an average of 50.0 years. More people had a young age of onset than an older age of onset. MPNST often also occurs in adults aged 20 to 50 years in other organs. The sex ratio was 4 to 12 in favour of women. The tumour diameter ranged from 2.5 to 17 cm. Our case was a young woman with a tumour diameter of 7.1 cm who had more of the common characteristics than the reported cases. Among the 16 cases, only four patients were diagnosed with MPNST before treatment. MPNST may be difficult to diagnose because the shape of the tumour resembles that of a submucosal tumour, which is also similar to oesophageal schwannoma; thus, MPNST is usually undiagnosable by routine biopsy. EUS-FNAB (endoscopic ultrasound-guided fine needle aspiration biopsy) and boring biopsy are useful for the diagnosis of submucosal tumours. In our case, because this patient had ulcer on the top of the protruding tumour, biopsy and diagnosis were easy. The macroscopic features of two cases were ulcers, and our patients had ulcers on top of the protruding type. The two patients with ulcers were diagnosed with MPNST, and one patient was diagnosed with a malignant tumour. In our case, we collected 6 biopsy samples from the tumour surface, around the ulcer and floor. Appropriate amounts of tumour cells were found only from the biopsy of the ulcer floor. The biopsies from the tumour surface detected necrotic tissue and fibroblasts. If we could not obtain an accurate diagnosis from the first biopsy, we would need a boring biopsy or EUS-FNAB. In these series, 8 patients underwent enucleation, and 7 patients underwent oesophagectomy. Because our patient was diagnosed with malignant disease before surgery, oesophagectomy was performed for to achieve radical resection. In this series of MPNSTs in the oesophagus, there was no recurrence in patients who underwent resection, including enucleation. However, many cases of local recurrence of MPNST in other organs have been reported. Nonetheless, when possible, we think that MPNST of the oesophagus should also achieve perfect local control. Two cases had distant metastasis, and these cases had a poor prognosis. On the other hand, the prognosis was good in cases in which radical resection was possible. Therefore, cases with distant metastases may require systemic treatment, such as chemotherapy. Few drugs have been shown to be effective, and treatment comprises the single agent doxorubicin or a combination of doxorubicin and ifosfamide [12]. Furthermore, with regard to malignancy, nerve cells normally abundantly express glucose transporter type 3 (GLUT-3), which transports for glucose into cells, and show high uptake during FDG-PET. It is difficult to evaluate the grade of malignancy from preoperative imaging tests. MPNST has an MIB-1 LI of 5 to 65%, whereas the MIB-1 LI for schwannoma is often 1% or less, which is useful for differentiation of benign versus malignant lesions.

**Table 1:** Reported cases of MPNST in the oesophagus

	Year	First author	Age (year)	Gender	Symptom	Tumour size (cm)	Macroscopic feature	Clinical diagnosis	Metastasis	Treatment	Recurrence	Prognosis (months)
1	2011	Wang	44	Female	Dysphagia	5.5 × 4.0 × 4.5	SMT	Leiomyoma	None	Enucleation	-	Alive (72)
2	2000	Manger	60	Female	Dysphagia, general fatigue, appetite loss, body weight loss	17	Protruding type	MPNST	None	Oesophagectomy	-	Alive (48)
3	2006	Basoglu	54	Female	Dysphagia, mass in neck	6 × 6	SMT	Not available	None	Oesophagectomy	-	Alive(40)
4	2010	Davydov	20	Female	Dysphagia	8.8 × 3.0 × 3.5	SMT	Not available	None	Enucleation	-	Alive(36)
5	1993	Iwata	56	Female	Abnormal shadow on chest X-ray	4.8 × 4.2 × 3.0	SMT	Leiomyoma	None	Enucleation	-	Alive (28)
6	1996	Morita	57	Female	Dysphagia	4.0 × 3.5 × 2.7	SMT	Not available	None	Enucleation	-	Alive (24)
7	2012	Teshima	64	Female	Abnormal shadow on chest X-ray	8.0 × 7.5 × 4.0	SMT	MPNST	None	Oesophagectomy	-	Alive (24)
8	2001	Murase	49	Female	None	8.2 × 5.8 × 3.7	SMT	Leiomyoma	None	Enucleation	-	Alive (27)
9	2002	Sato	55	Male	Dysphagia	8.5 × 7.0 × 4.0	SMT	Submucosal tumour	None	Enucleation	-	Alive (20)
10	2015	Mishra	27	Female	Dysphagia, palpitation, body weight loss	12 × 10 × 10	SMT	Leiomyoma	None	Oesophagectomy	-	Alive (18)
11	2012	Su	43	Male	Dysphagia	4.5 × 4.0 × 2.5	SMT	Not available	None	Enucleation	-	Alive (12)
12	2004	Sanchez	54	Male	Dysphagia	6 (gastroscopy report)	ulcer	MPNST	None	Oesophagectomy	-	Alive (2)
13	2003	Tsuji	49	Female	Dysphagia, cough	8.2 × 5.8 × 3.7	SMT	Leiomyoma	None	Enucleation	n/a	n/a
14	2010	Kitami	62	Male	Dysphagia, fever, body weight loss	Unknown	Irregularity	Non-small cell undifferentiated oesophageal cancer	Lymph nodes (cervical, mediastinum, left axillary, splenic hilum, para aorta, retroperitoneum)	Chemo-radiation		Death (12)
15	2015	Mavroeidis	76	Female	Dysphagia	3.9	Ulcer	Malignant tumour	Left adrenal gland, ribs, vertebras	Oesophagectomy chemotherapy		Death (5)
16	2018	Our case	31	Female	Dysphagia	7.1 × 6.1 × 5.5	Protruding type with ulcer	MPNST	None	Oesophagectomy	-	Alive (16)

*MPNST* malignant peripheral nerve sheath tumour; *SMT* submucosal tumour

Patients with neurofibromatosis type 1 have an approximately 10% probability of developing MPNST during their lifetime. In addition, approximately 50% of MPNSTs develop in patients with neurofibromatosis type 1. Treatment is based on extensive resection. Recently, a large cohort study showed that radiotherapy and chemotherapy were not associated with survival [13]. The treatment benefits of radiotherapy and chemotherapy for MPNST are unclear and controversial. MPNST is less responsive to radiation and chemotherapy, and surgical resection is central to its treatment. In addition, chemotherapy is indicated for large lesions and metastases. The most frequent sites of distant metastasis are the lungs, followed by the bones and the lymph nodes in the bones. Neurofibromatosis type 1 is not considered to be a risk factor involved in prognosis.

## Conclusion

Primary oesophageal MPNST is a relatively rare disease. Diagnosis before treatment is sometimes difficult, but the prognosis is good if radical resection is possible.

## Data Availability

Not applicable because this manuscript is a case report.

## Abbreviations

MPNST: Malignant peripheral nerve sheath tumour
EUS: Endoscopic ultrasonography
CT: Computed tomography
FDG-PET: 18F-fluorodeoxy glucose-positron emission tomography
SUV: Standardized uptake value
HPFs: High power fields
EUS-FNAB: Endoscopic ultrasound-guided fine needle aspiration biopsy
